# The Proposal to Erect the New Hospital on Clapham Common

**Published:** 1919-04-12

**Authors:** 


### WESTMINSTER HOSPITAL.

### The Proposal to Erect the New Hospital on Clapham Common.

### ARGUMENTS IN SUPPORT.

The following statements have been sent us of the grounds which the authorities hold to be important :

1. The site acquired by the Governors on Clapham Common is so generally recognised by everybody by whom it has been inspected as an ideal one for the purpose, whether regarded from the point of view of soil, aspect, elevation, accessibility, or surroundings, that it is unnecessary to labour the point.

2. It appears to be equally certain that a hospital is needed in this position. There is at present no large general hospital in the S.W. district south of the Thames, though the House of Lords' Committee of 1892, having specially in view the requirements of these districts, strongly recommended the moving of hospitals from the central portions of the Metropolis into those outside zones in which population is growing very rapidly.

There are, indeed, three comparatively small hospitals in the neighbourhood of the Clapham site; but these contain an aggregate of less than 160 beds, a number quite inadequate to provide for the hospital needs of the adjoining crowded and growing districts. In support of this contention the following facts may be mentioned :

(a) It is reported that at one of these three hospitals, viz., the South London for Women Only, there was recently a waiting list containing the names of as many as 300 persons, applicants for admission.

(b) At the last census in 1911 there was a population of upwards of 450,000 in parishes in the neighbourhood of Clapham Common, viz., Balham Hill, Battersea, Clapham, Earlsfield, Lavender Hill, Stockwell, Streatham, Tooting, and Wandsworth.

(c) A considerable proportion of the out-patients at present treated at Westminster Hospital come from districts on the south side of the Thames. In proof of this it may be stated that out of 205 new patients registered in the fourteen days from February 21 to March 6, 1919, 101 came from one or other of the following places, viz., Battersea, Clapham, Brixton, Balham, Streatham, Tooting, Wandsworth, Kennington, Stockwell, Vauxhall, Peckham, Dulwich, and Norwood. All of these patients would have found it easier to reach Clapham Common than Westminster.

3. Dealing with the question of finance and of the ability of the Governors to maintain a hospital at Clapham half as large again as the present hospital, it may be admitted at once that the only condition that would make future maintenance absolutely assured would be the possession of an endowment fund large enough to provide the whole annual income required. Westminster Hospital has been maintained, however, for the past 200 years without having ever been in this fortunate position, but has been supported for the most part by voluntary contributions, and such support, especially of late years, has been generously accorded. At the present time its total of invested funds stands at about £40,000 more than it did at the beginning of the present century, of which, speaking in round numbers, £29,000 is an addition to endowments, and £11,000 an addition to the funds available for general purposes. As regards the future also it appears to be highly probable that there will be new sources of revenue which have not existed in the past, and whether such revenue takes the form of subsidies from the State, or of capitation grants from the State or municipal authorities, it will in such case render the financing of hospitals in the future a less anxious task than in the past.

#### The Government's Views.

It would seem to be clear that in the future more hospital accommodation will be required than at present exists, and also that the voluntary hospitals will probably be conducted on somewhat different lines as is foreshadowed in remarks made in the House of Commons during the course of the Second Reading Debate upon the Ministry of Health Bill, when Dr. Addison spoke as follows :

"It was clear that there would be a wide extension of facilities in connection with nursing, midwifery, diagnosis, hospitals, and so forth. At present nobody suffered more than the poorly paid middle classes. Voluntary hospitals catered for the poor, and there was always a long waiting list. But the poor middle classes could not afford the fees of the great expert, and they did not want to be the recipients of charity."

#### A Working Member's View.

And Mr. J. R. Thomas, expressing the views of Labour, said :

"In order that the Bill should be effective large Exchequer grants to Local Authorities would be necessary in order that they might do their work. He knew the difficulty with the Treasury. But what might have been tolerated in 1914 was not good enough to-day. The people would not stand it. He would also like to see more provision in connection with the taking over of our voluntary hospitals, which ought to be national institutions provided by the public and administered by the public as a whole."

#### The Question of Amalgamation.

4. As regards the question of amalgamating Westminster Hospital with some similar institution or institutions, it is true that early in the year 1913 negotiations were set on foot for an amalgamation between Westminster and St. George's Hospitals. The proposal originated with St. George's, but it possessed attractions for Westminster, partly because it would have served to revert to the conditions which prevailed up to the year 1734, at which date the secession of St. George's took place, and partly because the resources of the two hospitals would have been sufficient to erect a new hospital of 500 beds and leave a sum of upwards of £900,000 for its maintenance. Though the negotiations referred to were carried on for some time, and even went so far as the drafting of a Bill for presentation to Parliament, the scheme ultimately broke down largely in consequence of the failure to agree upon matters of detail, a contingency which is always liable to occur in such cases.

#### The Question of the Medical School.

5. As touching the question of the Medical School, the following remarks are quoted from a statement recently issued to the Governors by Lord Glenconner, the Chairman of the Home Committee, viz. :

With regard to the effect which the carrying out of the Clapham scheme might have upon the fortunes of our Medical School, it is felt that the medical staff are somewhat unduly apprehensive. It may be pointed out that some years ago the school abandoned teaching the earlier subjects of the medical curriculum and arranged for our students to receive instruction in these subjects at King's College, a course also adopted by two other hospitals, and to this partial extent the number of medical schools in London has been diminished. Westminster students do not come to the hospital until they are ready to receive such instruction as can alone be given at the bedside of the patient.

What the policy of the Educational Authorities may be in the future cannot be foretold, but it would appear somewhat illogical to regard the substitution of an up-to-date hospital of 308 beds at Clapham for one of 213 beds at Westminster as a reason for withdrawing the recognition which the medical school at present enjoys. The fact of the school having done good work for the past seventy years would also seem to entitle it to every consideration.

There are those who consider that for some students it is better that they should be attached to a medical school which is not too large.

(*Concluded on p. 36 opposite.*)

### Westminster Hospital—(*concluded from p. 37, opposite*).

### THE HOPE OF THE 450,000 POPULATION AFFECTED.

6. The state of public local opinion upon the question of the establishment of the new Westminster Hospital on Clapham Common is clearly indicated in a letter from Sir Archibald Dawnay, who has been for many years past the Mayor of Wandsworth, and who writes as follows:

Borough of Wandsworth,
The Mayor's House,
4 Cedars Road, Clapham, S.W. 4,
March 20, 1919.

Westminster Hospital.

Dear Sir,—I am very pleased to have your letter hereon, it is a matter which I have had in mind very seriously and made it my business to get opinions upon, with the result that I believe the establishment of a hospital on the North Side of Clapham Common as proposed would be a magnificent gift to this part of South London; indeed, it will be a calamity if the scheme as suggested is not carried out in its complete entirety. There is no better or healthier site in the district for a hospital, and it is high time that the existing houses, however historic they may be, some of them, were cleared away and modern erections take their place; and I am sure if the district was polled an immense majority for the hospital would be the result. Pray do all you can to get the hospital erected, and I shall be glad to do anything which lays in my power to assist.—Very faithfully yours, (Signed) Archibald D. Dawnay, Mayor.

The Medical Officer of Health's Views.

Metropolitan Borough of Wandsworth,
Public Health Department,
79 East Hill, Wandsworth, S.W. 18,
March 21, 1919.

Dear Sir,—In reply to your letter of the 19th inst., I am strongly of opinion that the erection of a general hospital on North Side, Clapham Common, would be a very great boon to the inhabitants of this and neighbouring boroughs. At present in the borough of Wandsworth, with nearly 350,000 population, there is no general hospital. Previous to the war the hospital where the greatest number of deaths occurred of persons belonging to this borough was St. Thomas's, but ten times the number of deaths occurred in the Union Infirmaries. I have every reason to believe that a large proportion of the sick who at present go to the Union Infirmaries would preferably go to a general hospital if such a hospital existed nearer their homes.—Yours faithfully,
P. Caldwell Smith, Medical Officer of Health.

The Rev. E. A. Cartwright, Vicar of Christ Church, Battersea, writes:

"I think a new Westminster Hospital on Clapham Common would be a great boon to people in Battersea, though, I think, comparatively few attend that hospital from here. As far as my knowledge goes most go to St. Thomas's and St. George's, Women's Hospital, Chelsea, or Bolingbroke. For children, under the new Ministry, no doubt it would be very convenient, especially with the present tram service, which would take patients to the door"

